# Application of the Consolidated Framework for Implementation Research to assess factors that may influence implementation of tobacco use treatment guidelines in the Viet Nam public health care delivery system

**DOI:** 10.1186/s13012-017-0558-z

**Published:** 2017-02-28

**Authors:** Nancy VanDevanter, Pritika Kumar, Nam Nguyen, Linh Nguyen, Trang Nguyen, Frances Stillman, Bryan Weiner, Donna Shelley

**Affiliations:** 1 Rory Meyers College of Nursing, New York University, 433 First Avenue, New York, NY 10010 USA; 2 Department of Population Health, New York University School of Medicine, 227 East 30th St, New York, NY 10016 USA; 3 Institute of Social Medical Studies, The Institute of Social and Medical Studies, No 18 Lot 12B, Trung Yen 10 Street, Trung Hoa Ward, Cau Giay District, Ha Noi, Vietnam; 4 Johns Hopkins School of Public Health, 2213 McElderry Street, 4th floor, Baltimore, Maryland 21205 USA; 5 Department of Global Health, University of Washington, Harris Hydraulics Laboratory, Box 357965, Seattle, WA USA

**Keywords:** Smoking cessation, Tobacco use, CFIR, Qualitative methods, Vietnam

## Abstract

**Background:**

Services to treat tobacco dependence are not readily available to smokers in low-middle income countries (LMICs) where smoking prevalence remains high. We are conducting a cluster randomized controlled trial comparing the effectiveness of two strategies for implementing tobacco use treatment guidelines in 26 community health centers (CHCs) in Viet Nam. Guided by the Consolidated Framework for Implementation Research (CFIR), prior to implementing the trial, we conducted formative research to (1) identify factors that may influence guideline implementation and (2) inform further modifications to the intervention that may be necessary to translate a model of care delivery from a high-income country (HIC) to the local context of a LMIC.

**Methods:**

We conducted semi-structured qualitative interviews with CHC medical directors, health care providers, and village health workers (VHWs) in eight CHCs (*n* = 40). Interviews were transcribed verbatim and translated into English. Two qualitative researchers used both deductive (CFIR theory driven) and inductive (open coding) approaches to analysis developed codes and themes relevant to the aims of this study.

**Results:**

The interviews explored four out of five CFIR domains (i.e., intervention characteristics, outer setting, inner setting, and individual characteristics) that were relevant to the analysis. Potential facilitators of the intervention included the *relative advantage* of the intervention compared with current practice (intervention characteristics), awareness of the burden of tobacco use in the population (outer setting), *tension for change* due to a lack of training and need for skill building and leadership engagement (inner setting), and a strong sense of *collective efficacy* to provide tobacco cessation services (individual characteristics). Potential barriers included the perception that the intervention was more *complex* (intervention characteristic) and not necessarily *compatible* (inner setting) with current workflows and staffing historically designed to address infectious disease prevention and control rather than chronic disease prevention and competing priorities that are determined by the MOH (outer setting).

**Conclusions:**

In this study, CFIR provided a valuable framework for evaluating factors that may influence implementation of a systems-level intervention for tobacco control in a LMIC and understand what adaptations may be needed to translate a model of care delivery from a HIC to a LMIC.

**Trial registration:**

NCT02564653. Registered September 2015

## Background

Tobacco use is the leading cause of preventable death globally. Nearly 80% of the more than one billion smokers worldwide live in low- and middle-income countries (LMICs), where the burden of tobacco-related illness and death is heaviest [1, 2]. In Viet Nam, 45% of men use tobacco, which is one of the highest prevalence rates in the world [3]. In 2004, Viet Nam took a significant step toward furthering its tobacco control efforts by ratifying the World Health Organization’s (WHO) Framework Convention on Tobacco Control (FCTC), an evidence-based global public health treaty developed by the WHO in response to the globalization of the tobacco epidemic [4]. Since ratifying the treaty, Viet Nam has made significant progress in implementing several components of the FCTC, including smoke-free air laws and counter advertising campaigns [5]. However, the FCTC also requires parties to implement effective strategies to promote cessation and provide adequate treatment for tobacco dependence (Article 14) [6]. Viet Nam recently launched a national quitline. However, treatment for tobacco use is not integrated into the health care delivery system, and therefore, patients are not routinely screened or offered evidence-based assistance [7]. This is in part due to a lack of training, funding, and infrastructure to support these activities and a lack cost effective strategies for implementing evidence-based approaches to treating tobacco use in the context of public health systems in LMICs [7–10].

A recent assessment of research priorities to support the successful implementation highlighted this gap and concluded that there is a need to study new models of tobacco use treatment that addresses multilevel barriers to implementing Article 14 guidelines in low-resource health settings [8]. There is evidence for several strategies that enhance smoking cessation adoption in clinical settings including clinical reminder systems, provider feedback, and referral systems that allow providers to delegate follow-up and additional counseling [11–13]. However, policy and system approaches that are effective in high-income health systems are likely to require adaptations to achieve similar results in low-resource public health care delivery settings in LMICs [14, 15].

We are conducting a cluster randomized controlled trial (RCT) that is comparing two multilevel, multicomponent strategies to increase implementation of tobacco use treatment guidelines in community health centers (CHCs) in Viet Nam [16]. The control sites receive training, a tool kit with patient and provider materials, and a reminder system to prompt screening and brief counseling (TTR). The intervention sites receive TTR plus a system to refer patients to a village health worker (VHW) for more intensive cessation counseling. Prior to implementing the intervention, we conducted a formative assessment to identify potential barriers and facilitators to implementing system changes to increase adoption of tobacco use treatment guidelines in this context and to inform modifications to optimize translation of a model of care delivery that has demonstrated effectiveness in high-income countries (HICs) but has not been tested in a LMIC delivery system.

The Consolidated Framework for Implementation Research (CFIR) guided the formative assessment [17]. CFIR is a meta-theoretical framework that offers an overarching typology for delineating barriers and facilitators influencing implementation and to assist in understanding “what ‘works where and why’ across multiple contexts” [17, 18]. CFIR comprises 39 constructs under five major domains: (1) intervention characteristics, (2) outer setting, (3) inner setting, (4) individual characteristics, and (5) process. The domains and constructs represent a synthesis of a range of theories about dissemination, innovation, and implementation.

A recent systematic review of the use of CFIR identified 26 studies that met eligibility criteria [19]. Only two studies applied the model in the preimplementation phase. It is surprising that so few of the studies have applied this framework preimplementation given the complexity of moving evidence-based guidelines into practice and the need to tailor these guidelines to the local practice context [14, 20]. This paper presents findings that provide insights into the multilevel factors that may facilitate or impede guideline implementation in health systems in LMICs and how formative research, guided by a conceptual framework for implementation, can guide adaptations needed to tailor implementation strategies to local context.

## Methods

### Study design

We conducted a qualitative study consisting of semi-structured in-depth interviews with 40 health care providers and VHWs prior to implementing a two-arm cluster RCT. The RCT is comparing the effectiveness of two strategies for implementing tobacco use treatment guidelines in 26 randomly selected CHCs in Thai Nguyen, Viet Nam, a rural province north of Hanoi. Sites are enrolled in the intervention in three waves (eight in wave 1, ten in wave 2, and eight in wave 3). The interviews were conducted with medical directors, health center staff, and VHWs in each of the eight CHCs enrolled in the first wave. Details related to site selection are described in a previous article [16].

### Study setting

The Vietnamese health care system is hierarchically organized into four administrative levels: central, province, district, and community. The CHCs serve as the primary access point for public health and preventive care services in Viet Nam, each providing services for an average of 5000–7000 people in their surrounding community. Each CHC is staffed by five to six clinicians, including one physician and three to five other health professionals (e.g., nurses, midwives). In addition, each CHC is supported by a network of eight to ten community health workers, referred to as village health workers (VHWs) in Viet Nam. VHWs’ primary responsibility is to implement the national health promotion and prevention priorities in their communities. In addition, they serve as a bridge between the community and their local CHC.

### Sample and recruitment

We recruited a purposely selected sample of health care providers to achieve representation across all levels of staff. This included one medical director, two CHC staff members, and two VHWs from each of the eight CHCs. We obtained informed consent from all participants consistent with the procedures approved by the New York University School of Medicine and Institute for Social Medical Studies Institutional Review Boards. The interviews were conducted in Vietnamese, lasted approximately 1 h, and were audiotaped. All interviews were transcribed in their original language and were translated by professionals’ fluent in Vietnamese and English.

### Data collection and measures

The interview guides were pretested with providers from CHCs that were not participating in the study and covered the following topics within four CFIR domains: (1) intervention characteristics (e.g., current practices for treating tobacco use, relative advantages or disadvantages of the proposed intervention components over current practice, and suggested adaptations); (2) outer setting (e.g., perceived need for services for tobacco cessation in the community, role of the Ministry of Health (MOH) policies in driving which services were implemented in these settings); (3) inner setting (e.g., perceptions about leadership engagement, relative priority of tobacco use, compatibility of the proposed intervention, and tobacco use treatment in general, with current workflows and staffing resources, experiences with various health care programs run by their respective CHCs, and their reports of organizational factors affecting the implementation of past public health programs); and (4) individual characteristics of providers (e.g., knowledge, attitudes and beliefs about the health effects of tobacco use, and self-efficacy to offer treatment).

### Data analysis

Two members of the research team (NV and PK) conducted the thematic content analysis. Initially, each researcher read the transcripts independently identifying preliminary codes and subthemes using both an inductive and deductive approaches (open coding and coding of theoretical constructs). Coding differences between the primary coders were resolved by discussion among the larger research team members by going back to the original transcripts and consulting with the field researchers. Major themes were then mapped to the domains and constructs of the CFIR. The transcripts were coded utilizing Atlas ti software. A random sample of 20% of transcripts was independently coded by another member of the research team to establish inter-rater reliability (Kappa 0.80).

## Results

We present findings within each of the four CFIR domains evaluated.

### Intervention characteristics

Most participants perceived the multicomponent intervention as a *relative advantage* compared with the current practice in which patients were not routinely screened or offered tobacco cessation counseling. Almost all of the participants noted that a major reason why they did not offer treatment was the lack of training among the clinicians and staff. The training was therefore viewed as a particularly valuable component of the proposed intervention: “The program against cigarette smoking is a new one. Never have health care providers been trained. We want to get trained properly.” (CHC staff # 4).

Another *relative advantage* of the proposed intervention model that emerged from the data was the integration of VHWs as a referral resource. VHWs were described as having tremendous credibility among community members. One CHC staff participant suggested: “Your program should depend on village health workers because they are closer to the people in the community compared to Community Health Center Staff and Health Care Providers.” (CHC staff # 16). Another CHC staff member noted: “They are influential in their village. In other words, their speech should have some power in their village. VHWs are close to people in every village.” (CHC staff # 12).

A potential barrier that emerged was the perceived complexity of offering counseling and advice to quit which they believed would take more time compared with their current focus on prevention and treatment of infectious diseases: “…our working schedule is very tight and the workload is very heavy. We don’t have much time to talk to or advise patients” (CHC Director #4). This theme emerged again in discussing the intervention’s compatibility with current workflow, which is described below under the inner setting domain.

### Outer setting

*Patient needs* were identified as an important *outer setting construct* that could drive demand for services and facilitate clinicians’ support for implementing the intervention. Almost half of the participants described a high degree of community concern about the health effects of tobacco: “People in the community do not agree with smoking anymore because they know that smoking is directly harmful for the health of smokers and indirectly for the health of surrounding people” (CHC staff #7). Many participants expressed the belief that the serious health consequences of tobacco use for the community increased the *relative priority* of the program. “It is unreasonable to overlook this while smoking is the root of many diseases” (CHC Director #3).

Tobacco control *policies* (outer setting construct) have raised awareness of the harmful effects of tobacco use. However, the MOH policies have not included resource allocations to support a role for CHCs in providing cessation services. Several CHC directors acknowledged that the MOH is prioritizing tobacco control but there is no “grassroots” support for CHCs: “I have not seen any programs. I assume that there are more urgent issues for the MOH so this issue has not received equal attention”. (CHC Director # 6). This was perceived as a potential barrier to sustaining new programs, a challenge they have experienced with previous projects funded through nongovernmental sources. However, because of the expressed need for training and resources, the lack of current MOH support did not seem to diminish interest in participating in the study intervention which was viewed as an important resource for growing staff capacity to meet smokers’ needs: “Currently we do everything without support. With this program we are equipped with knowledge… together with our willingness to participate the rate of success will be higher” (CHC Director #2).

### Inner setting

Several subconstructs of *implementation climate*, a key construct of the inner setting domain, were identified as potentially relevant to the proposed intervention. This included *tension for change*, *relative priority*, *learning climate*, and *compatibility*. Participants again referenced their lack of training in tobacco cessation, but in this context, it was described as contributing to a *tension for change*. One CHC staff member described the consequences of staffs’ lack of knowledge about tobacco cessation: “One can only provide patients basic knowledge, something that they might know already. We have no profound knowledge to give them further advice. In order to do that, we need to get the proper training.” (CHC staff #6).

Although tobacco use was consistently described as an important issue, a potential barrier to implementation of the intervention was the numerous competing programs that the MOH mandates. For some, these prevention programs remained the priority compared with adding new responsibilities to offer tobacco use treatment. One participant noted: “Certainly, other health care services are more important than this smoking cessation program. We are running over 10 programs.” (CHC Staff #15).

Another potential and related barrier to implementation was concerns about the *compatibility* of integrating tobacco use treatment into their existing workflow. Given how busy they are with other priority programs, several participants noted that adding yet another clinical intervention would pose challenges to keeping up with their current workloads. In addition, concerns about compatibility seemed highly related to concerns about fitting an intervention that seemed more *complex* (intervention characteristic domain) than their typical clinical interactions with patients into an already busy schedule. Specifically, one staff member observed that a shift from focusing on preventing infectious disease to prevention of chronic disease may result in increasing demands on staff time and resources. As one medical director described it: “Normally we offer treatment and make prescription, but now we have to offer consultation as well.” (CHC Director #8). However, the proposal to delegate more intensive counseling to VHWs (ARM 2 of the study design) was viewed by one staff as a way to alleviate some of these concerns: “VHWs can do the work better because they have more time…they live in the same village as patients so they can spend their time counseling at any time.” (CHC staff #3).

Two factors were identified as potentially facilitating implementation by contributing to a positive *learning climate*. The majority of participants cited the successful implementation of other MOH priority programs as evidence of their ability to effectively implement the tobacco cessation program: “Because of all programs which were already implemented here were successful. Therefore, I think that the same situation will happen to this program.” (CHC staff #5). In addition, the majority of participants described positive outcome expectations of staff that also contributed to a positive learning climate and increased potential for successful program implementation: “I’m sure we can do it. Enthusiasm and knowledge are the keys to successful health care. We have great enthusiasm. If we also have knowledge, there is nothing we cannot do.” (CHC staff #13). This statement also reflected a sense of collective efficacy that emerged in questions related to individual characteristics.

*Leadership engagement*, an inner setting construct related to readiness for implementation, emerged as another potential facilitating factor. “From the time we were informed about this program by the CHC director, all CHC staff and health care providers are serious toward the implementation of this program” (CHC staff #11).

### Characteristics of individuals

Consistent with health care workers’ lack of personal autonomy and the collective nature of the Viet Nam culture, *collective efficacy* emerged as more essential than *self-efficacy* as a potential facilitator of program implementation. This was most apparent in the use of “we” rather than “I” when describing their confidence in implementing the program, which is evidenced by these quotes from VHWs and CHC staff: “We try our best” (VHW #1). “We will exert great efforts to ensure success.” (VHW #3) “We are confident we can counsel people about giving up smoking.” (CHC staff #1) “If we get training properly, along with great experience, we will be able to deliver better results.” (CHC staff #7).

Another aspect of collective efficacy that seemed to overlap with the inner setting constructs of culture and climate was the frequently articulated respect for their colleagues and confidence in their capacity to contribute equally to patient and community health, regardless of discipline and education level: “VHWs and CHC staff are in charge of different tasks and we support each other in each task” (CHC Director 1). “To me, they are like my long arm and whether health care at grassroots levels is good or not mainly depends on village health workers” (CHC Director #4).

Participants did not specifically reference *individual identification with the organization*, a broad construct related to how individuals perceive the organization and their degree of commitment to that organization. Rather similar to the construct of efficacy, identification with the organization was expressed as a sense of pride in their particular center’s collective capacity to implement MOH-driven programs, despite, at times, a lack of resources to support their work. A CHC staff member noted: “I believe if we get training properly, along with great experience we will be able to deliver better results.” (CHC staff #9).

### Overlap of outer and inner setting domains

Viet Nam’s culture and political structure create a blurring of the lines between outer and inner setting domains. In Viet Nam, public health goals and priorities are set top down, with the MOH and district health leaders setting the agenda for local community-level public health programs. Therefore, CHCs and their staff have limited involvement in decisions about the design of new interventions and initiatives. As a VHW explained: “The program (intervention) will certainly meet many difficulties if it is not a priority of the Ministry of Health.” (VHW #6). The *tension for change* and *relative priorities* (*inner setting subconstructs*) were also driven by outer setting or external factors including perceptions about the extent of the public health problem and whether or not the MOH would explicitly include tobacco use treatment as a priority prevention program and provide resources to support program implementation.

## Discussion

The CFIR framework provided a valuable structure for identifying, across four domains, interrelated facilitators and barriers to implementing a multicomponent intervention to increase adoption of tobacco use treatment guidelines. In applying this framework to the analysis, we found a number of potential facilitators including the high value that CHC health care providers, staff, and VHWs placed on the training component of the intervention and infrastructure and capacity building opportunities that participation in this program offered. This high level of interest seemed, in part, related to the MOH’s tobacco control activities that are increasing awareness of the consequences of tobacco use. At the same time, raising awareness about the consequences of tobacco use has highlighted gaps in capacity among CHC providers who do not have the expertise or resources to treat nicotine addiction. Several surveys of providers in LMICs, including Viet Nam, have described gaps in training as a significant barrier to provider adherence to recommended tobacco use treatment [7]. This was echoed in our interviews and informed some modifications to the intervention, which included lengthening the initial training session and adding a booster session to provide opportunities to reflect on experiences and continue to build capacity.

Despite enthusiasm for the intervention, some providers raised concerns about competing priorities that are determined by the central government and not locally and the compatibility of adding a new program to an already heavy workload, particularly one that may require more complex and time-consuming interactions with patients than their current prevention priorities require. In discussing challenges to treating tobacco use, it became clear that the growing burden of noncommunicable diseases (NCDs), largely caused by tobacco use, may require rethinking roles and responsibilities and restructuring the health care delivery team. The proposed intervention, which was designed to test the effectiveness of task shifting more intensive cessation counseling to the VHWs, provides an example of a promising staffing model to address the demands of chronic disease prevention and treatment [21].

Task shifting health care responsibilities from physicians or allied health professionals to lay health care workers has been endorsed by the World Medical Association and is a strategy that may help meet the global challenge of the growing NCD epidemic in low-resource settings [22]. There was enthusiasm for the described intervention model that was viewed as both reducing provider burden and leveraging the respect and credibility. VHWs have in their communities and their established role in providing community-based preventive services. Focus groups with VHWs and surveys with providers in Viet Nam in a previous study also described this role as a good “fit” with their current responsibilities [7, 23].

Important cultural differences emerged that had the potential to impact implementation effectiveness. In the collectivist social model prevalent in Viet Nam, individual efficacy was rarely discussed; however, collective efficacy, when individuals in a community share values, have trust, solidarity, and operate cooperatively, was prominent in the narratives. There is evidence that outcomes desired by a community are more likely to occur where there is collective efficacy [24]. Therefore, it is worth exploring further how collective efficacy may be strengthened around tobacco use treatment and prevention to increase adoption of recommended care. This finding did lead to modifications in the training format. For example, we originally planned to train the VHWs separately but decided to integrate them into the CHC health care worker training after which they received an additional 3 days of training on the intensive three session counseling intervention component.

Other cultural and political characteristics of Viet Nam emerged as potential barriers to sustainability more than short-term implementation. For example, the centralization of decision-making authority within the Viet Nam MOH with regard to health priorities lent greater weight to the outer setting constructs than to the inner setting and created a blurring of the inner and outer setting domains in this context. Damschroder and colleagues anticipated this finding stating that “the line between inner and outer setting is not always clear and the interface is dynamic and somewhat precarious as specific factors considered in or out will depend on the context” [17]. In Viet Nam, CHC staff and VHWs lack autonomy to implement programs other than those authorized by the MOH and center resources are aligned with MOH priorities and depend on MOH allocations. This makes it difficult for CHCs to innovate locally and to sustain interventions without MOH support.

The dominance of the MOH in determining the public health care system prevention and treatment agenda required early and ongoing engagement with MOH leadership during the grant development phase to facilitate implementation. The interview findings, however, provided additional community-level insights about how the political and organizational cultural of the public health system in Viet Nam might create potential barriers to sustainability and scale-up, if the intervention was effective. This prompted additional efforts to align the project with national tobacco control priorities for implementing Article 14 and plan for sustainability and wider dissemination throughout the public health system. These efforts included establishing an external advisory board with members representing a broad array of key stakeholders, including the MOH’s Director of the Viet Nam Steering Committee on Smoking and Health (VINACOSH), ongoing one-on-one meetings with VINACOSH, and membership of local investigators from the research team on the VINACOSH tobacco cessation workgroup.

As our findings indicate, the use of theoretical models to assess implementation and dissemination processes and outcomes can contribute to greater precision in intervention design and implementation, particularly in LMICs that lack experience implementing organizational and system changes to optimize guideline implementation [17, 18]. Based on a recent systematic review of the use of CFIR, most studies have applied the framework to assess barriers and facilitators to innovation implementation in the postimplementation phase [19]. Robins et. al.’s study, one of the two studies included in the review that applied the CFIR in the preimplementation phase, sought to identify contextual barriers and facilitators to implementation a home-based blood pressure monitoring program in primary care practices [25]. The US setting and differences in the intervention design may make it difficult to draw comparisons; however, there were some similar issues that emerged including concerns about time demands associated with the new intervention (inner setting). At the same time, staffs were similarly enthusiastic about an approach that would support providers to do a better job helping patients to control their blood pressure (relative advantage of intervention over current practice). A notable difference between our findings and theirs was skepticism about the need to collaborate with other health professionals to implement a new team care model. This was in contrast to the confidence expressed by Vietnamese CHC providers and staff in collaborating with VHWs and creating a team approach to tobacco use treatment. This, again, may reflect the predominance of collective efficacy in the Vietnamese context vs. individual self-efficacy in the US context.

The only study in the review conducted in a LMIC (Kenya) and also in the preimplementation phase, combined interviews with key stakeholders and a review of review of implementation science theories, including CFIR, to inform their intervention design. The authors did not describe an assessment of CFIR constructs but rather planned characteristics of the intervention design that was linked to CFIR constructs (e.g., development of a learning climate, leadership engagement, enhancing self-efficacy). English did describe a similar need to engage multiple stakeholders in developing guidelines and a process that was “undertaken with the authority of the government” [15].

Through cultural interpretations, it is possible to deliver interventions that are contextualized in terms of cultural values, language, and socio-economic status and personal preferences. Understanding cultural differences in how behavior is influenced may improve adoption of guideline-recommended care in different settings and wider implementation of Article 14 in LMICs.

There are several limitations to the study. The qualitative interviews were conducted in eight of the 26 CHCs and may not reflect differences across other clinics in the region. In addition, the CHCs are all located in rural areas of North Viet Nam and may not reflect the environment in other parts of the country, though the centralized nature of the health system makes it likely that the same challenges would be found. The study was conducted prior to the implementation of the intervention training and implementation, so we were not able to assess all of the constructs of the CFIR. Postintervention interviews assessing the CFIR variables will be critical to assessing the predictive value of the baseline assessment.

## Conclusions

Globally, public health priorities are shifting to deal with the growing burden of tobacco use and other NCDs. However, resources have not been allocated to build capacity at the local level to address the significant burden of tobacco-related illness. Our assessment pointed to several potential facilitators and barriers to implementing a new model of collaborative care that includes VHWs to optimize integration of evidence-based tobacco use treatment in CHCs. In summary, potential facilitators of the intervention included the *relative advantage* of the intervention compared with current practice, MOH-driven *policies* that support tobacco control and growing awareness of the burden of tobacco use in the population, *tension for change* due to a lack of training and a recognized need for skill building, and *leadership engagement* and a strong sense of *collective efficacy* to provide tobacco cessation services if they received the proper training and resources. Potential barriers included the perception that the intervention was more *complex* and not necessarily *compatible* with current workflows and staffing that were historically designed to address infectious disease prevention and control rather than chronic disease prevention, *competing priorities* that are determined by the MOH, and a lack of *resources* coming from the MOH to support a sustainable model for integrating tobacco use treatment into routine care. However, a model that shifted primary responsibility for counseling to VHWs was viewed as consistent with their role and an appropriate solution to reducing clinician burden. Given the top down model of the health care delivery system in a country like Viet Nam, it is important to engage key stakeholders at national, provincial, and district level (not just communal level) when building sustainability mechanisms for NCD programs, including tobacco use treatment. Cultural adaptations are possible and most relevant through formative evaluation driven by comprehensive implementation frameworks like CFIR that allow for flexible interpretations and adaptation.

## Abbreviations

CFIR: Consolidated Framework for Implementation Research
CHC: Community Health Centers
MOH: Ministry of Health
NCDs: Noncommunicable diseases
VHW: Village health worker
